# Anti-RANKL Inhibits Thymic Function and Causes DRONJ in Mice

**DOI:** 10.1155/2022/9299602

**Published:** 2022-04-15

**Authors:** Yusuke Nakamura, Takashi Kikuiri, Takahiro Sugiyama, Ayako Maeda, Daisuke Izumiyama, Daigo Yahata, Yoshitaka Yoshimura, Tetsuo Shirakawa, Yoshimasa Kitagawa

**Affiliations:** ^1^Department of Oral Diagnosis and Medicine, Faculty of Dental Medicine and Graduate School of Dental Medicine, Hokkaido University, Sapporo 060-8586, Japan; ^2^Department of Pediatric Dentistry, Nihon University School of Dentistry, 1-8-13 Kanda-surugadai, Tokyo 101-8310, Japan; ^3^Department of Dentistry for Children and Disabled Person, Faculty of Dental Medicine and Graduate School of Dental Medicine, Hokkaido University, Sapporo 060-8586, Japan; ^4^Department of Oral and Maxillofacial Surgery, Faculty of Dental Medicine and Graduate School of Dental Medicine, Hokkaido University, Sapporo 060-8586, Japan; ^5^Department of Pharmacology, Faculty of Dental Medicine and Graduate School of Dental Medicine, Hokkaido University, Sapporo 060-8586, Japan

## Abstract

**Background:**

Denosumab, a human monoclonal antibody against receptor activator of nuclear factor-kappa B ligand (RANKL), is a novel bone antiresorptive agent used in patients with osteoporosis or metastatic bone cancer. Denosumab-related osteonecrosis of the jaw (DRONJ) has been recently reported in patients using denosumab. However, the mechanisms of DRONJ are not fully understood. Appropriate pathogenic mechanisms of DRONJ have yet to be established. Therefore, we investigated the pathogenesis of DRONJ in mice.

**Methods:**

Anti-mouse RANKL monoclonal antibody and melphalan were performed to create a mouse model of DRONJ-like lesions in female C57BL/6J mice. We examined the development of DRONJ-like lesions and immune function.

**Results:**

We showed that administration of anti-mouse RANKL monoclonal antibody and melphalan caused DRONJ-like lesions that recapitulated major clinical manifestations of the human disease, including the characteristic features of an open alveolar socket and exposed necrotic bone. In the analysis using a mouse model of DRONJ-like lesion, it was revealed that anti-mouse RANKL monoclonal antibody and melphalan suppress autoimmune regulator (AIRE) expression in the thymus and imbalanced T cell populations.

**Conclusion:**

This study suggests evidence of an immunity-based mechanism of DRONJ-like disease. This work may contribute to a better understanding of the pathogenesis of human DRONJ.

## 1. Introduction

Antiresorptive medications are currently being used to treat bone fragility, such as in osteoporosis. The discovery of receptor activator of nuclear factor-kappa B (RANK) and its ligand, the RANKL pathway, has led to the development of a new therapeutic agent for osteoporosis [1]. Denosumab is a human monoclonal antibody of RANKL and binds to the RANK-binding sites to inhibit osteoclast development [2]. Denosumab-related osteonecrosis of the jaw (DRONJ) has been reported in patients treated with denosumab [3]. DRONJ is very severe, but there are no currently recommended treatments. It is important to clarify the pathogenesis of DRONJ in order to develop an effective approach for treating and preventing it. We therefore hypothesized that abnormal RANKL signals are responsible for the development of DRONJ. It has been reported that RANKL is also a crucial cytokine for forming immune tissues such as the thymus and lymph nodes [4]. The present study on the association between DRONJ development and immune function was validated using mice.

## 2. Methods

### 2.1. Animals

This study used C57BL/6J mice (4 weeks-old female; Sankyo Laboratory, Sapporo, Japan). All animal experiments were performed under an institutionally approved protocol for the use of animal research at Hokkaido University (No.15–0015). Mice were kept in animal facilities under controlled temperatures and humidity, and under a 12-hour light/dark cycle. Food and filtered water were provided ad libitum.

### 2.2. Mouse Model for DRONJ-Like Lesion

**Figure 1:**
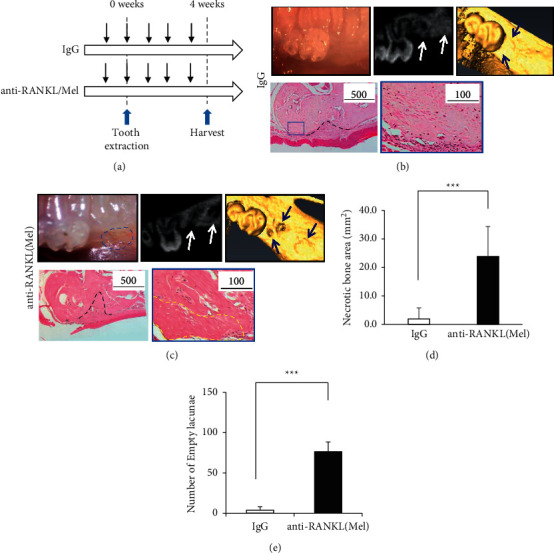
Development of a mouse model of DRONJ-like lesions. (a) The schematic timeline protocol for a mouse model of DRONJ-like lesions. (b) The upper left panels show the representative gross appearance of gingival mucosa at the extraction site of IgG. The upper middle panel shows *μ*CT scans of the extraction site. White arrows indicate extraction sockets of the root with new bone formation. The upper right panel shows a 3D image of the extraction site. Blue arrows indicate extraction sites filled with new bone formation. The lower left panel shows H&E staining of a low magnification image at the extraction site. A black dotted line surrounds the outline of the extraction socket of the root. Bar is 500 *μ*m. The lower right panel shows H&E staining of a high magnification image of the blue box in the lower left panel. Bar is 100 *μ*m. (c) The upper left panels show the representative gross appearance of gingival mucosa at the extraction site of anti-RANKL/Mel. The blue dotted line indicates the open socket of the extraction site. The upper middle panel shows *μ*CT scans of the extraction site. White arrows indicate extraction sockets of the root without new bone formation. The upper right panel shows a 3D image of the extraction site. Blue arrows indicate extraction sockets of the root without new bone formation. The lower left panel shows H&E staining of a low magnification image at the extraction site. A red asterisk indicates an open socket with an area of exposed bone and no mucosal coverage at the extraction site. A black dotted line indicates the outline of the extraction socket of the root. Bar is 500 *μ*m. The lower right panel shows H&E staining of a high magnification image of the blue box in the lower left panel. A yellow dotted line indicates a necrotic bone area with an empty lacuna. Bar is 100 *μ*m. (d) Necrotic bone areas in IgG vs. anti-RANKL/Mel. E: number of empty lacunae in IgG vs. anti-RANKL/Mel. Graphs show mean ± SEM. ^*∗∗∗*^*p* < 0.001.

Denosumab is a human monoclonal antibody against RANKL and has no affinity in mice; therefore, we used an anti-mouse RANKL monoclonal antibody (anti-RANKL; Oriental Yeast Co., Ltd., Tokyo, Japan) to induce DRONJ-like lesions in mice. For the DRONJ-like lesion mice (*n* = 7), 1.0 mg/kg of anti-RANKL and 7.0 mg/kg of melphalan (Mel; GlaxoSmithKline, Tokyo, Japan) were administered intravenously through the tail vein once a week. The control mice (*n* = 7) received intraperitoneal injections of 1.0 mg/kg of control rat IgG monoclonal antibody (FUJIFILM Wako Pure Chemical Corporation, Tokyo, Japan) once a week. One week after injection, maxillary first molars were extracted under general anesthesia using pentobarbital (Kyoritsu Seiyaku Corporation, Tokyo, Japan). Four weeks after teeth extraction, mice were harvested using carbon dioxide, CO_2_ (Figure 1(a)).

### 2.3. Microcomputed Tomography (*μ*CT)

Maxilla specimens were scanned using *μ*CT (LaTheta LCT-200; Hitachi Aloka Medical, Ltd., Tokyo, Japan) at a 24 *μ*m voxel resolution with an energy level of 50 kV.

### 2.4. Histology Analysis

After *μ*CT scanning, the maxilla was decalcified in 10% ethylenediaminetetraacetic acid (EDTA) in PBS (pH 7.4). The maxilla and thymus were embedded in paraffin following dehydration in ethanol. Samples were cut into 8 *µ*m sections and hematoxylin and eosin (H&E) staining was performed.

### 2.5. Immunohistochemistry

Deparaffinized sections of the thymus were washed, and endogenous peroxidase activity was quenched by immersion in 3% hydrogen peroxide in methanol. Sections were then incubated with antiautoimmune regulator (AIRE) antibodies (1 : 200 dilution, Sigma-Aldrich, St. Louis, MO, USA) for 1 h. Isotype control antibodies (Sigma-Aldrich) were used under the same conditions. A SuperPicTure Polymer Detection Kit (Zymed/Invitrogen, Carlsbad, CA, USA) was used for enzymatic immunohistochemical staining in accordance with the manufacturer's protocols.

### 2.6. Terminal Deoxynucleotidyl Transferase (TdT)-Mediated dUTP Nick End Labeling (TUNEL) Assay

A TUNEL Assay Kit (HRP-DAB) (Abcam, Cambridge, UK) was used to detect apoptotic cells in the thymus according to the manufacturer's instructions. For each group, the number of positive cells in randomly chosen high-power fields in the cortex and medulla areas was counted using a light microscope.

### 2.7. Flow Cytometry

Thymocytes, peripheral blood mononuclear cells (PBMCs), and splenocytes were analyzed by flow cytometry. Thymocytes and PBMCs were stained with fluorescein isothiocyanate (FITC)-conjugated anti-CD4 (BioLegend, San Diego, CA, USA) and phycoerythrin (PE)-conjugated anti-CD8 antibody (BioLegend). Splenocytes were stained with FITC-conjugated anti-CD4 and allophycocyanin (APC)-conjugated anti-CD25 (BioLegend), fixed, and permeabilized using the FOXP3 Fix/Perm Buffer Set (BioLegend) and stained with PerCP-Cy5.5-conjugated anti-FOXP3 (BioLegend) according to the manufacturer's protocol. Samples were run using FACS Canto (BD Biosciences PharMingen, Franklin Lakes, NJ, USA).

### 2.8. Statistical Analysis

The results were expressed as the means ± SD. Overall comparisons were made using ANOVA, and statistical differences between the groups were determined by Bonferroni's test. Data with a *P*value <0.05 were considered significant.

## 3. Results

### 3.1. Anti-RANKL/Mel Administration Enhance DRONJ-Like Lesion in Mice

The preliminary experiment examined whether a single administration of anti-RANKL and Mel caused DRONJ. The results revealed that all mice with single-administrated anti-RANKL and Mel did not develop DRONJ-like lesions, such as failure to wound heal with normal mucosal or bone formation (Appendix Table and Appendix Figures 1–3). Clinical findings at the tooth extraction site showed complete closure of the epithelium in 100% (7 of 7) of the control rat IgG monoclonal antibody administered mice (IgG) (Figure 1(b)). In contrast, in both anti-RANKL and Mel administered mice (anti-RANKL/Mel), 42.9% (3 of 7) of mice had exposed bone without complete closure on the epithelium (Figure 1(c). The *μ*CT scanning at the tooth extraction site confirmed that IgG was filled with new bone in the socket. In anti-RANKL/Mel, there was an unabsorbed alveolar ridge and an absence of osteogenesis within the socket (Figure 1(c)). Furthermore, histological examination revealed that IgG had epithelial coverage of the extraction site and no exposed bone (Figure 1(b)). On the other hand, typical findings of osteonecrosis of the jaw, such as breakage of oral mucosal tissue, absence of connective tissue layers, and empty lacunae, were identified at the extraction site in anti-RANKL/Mel (Figure 1(c)). The area of osteonecrosis of the jaw with empty lacunae was 1.9 ± 3.9 mm2 in IgG and was significantly higher in anti-RANKL/Mel with 23.9 ± 10.5 mm2 in anti-RANKL/Mel (*P* < 0.001) (Figure 1(d)). Also, the empty lacunae count was significantly higher (*P* < 0.001) in anti-RANKL/Mel compared to IgG (76.5 ± 11.9 vs. 4.0 ± 4.0, respectively) (Figure 1(e)).

### 3.2. Anti-RANKL/Mel Induced the Suppression of Immunity and the Dysfunction of Immunotolerance

**Figure 2:**
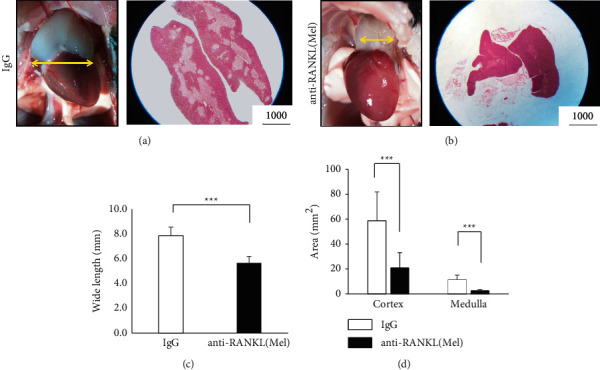
Anti-RANKL/Mel administration causes atrophy of the thymus. (a) The left panel shows the representative gross appearance of the thymus in IgG. Yellow arrows indicate the wide length of the thymus. The right panel shows H&E staining of a low magnification image of the thymus. Bar is 1000 *μ*m. (b) The left panel shows the representative gross appearance of the thymus in anti-RANKL/Mel. Yellow arrows indicate the wide length of the thymus. The right panel shows H&E staining of a low magnification image in the thymus. Bar is 1000 *μ*m. (c) Wide length of the thymus in IgG vs. anti-RANKL/Mel. (d) Area of the cortex and medulla of the thymus in IgG vs. anti-RANKL/Mel. Graphs show mean ± SEM. ^*∗∗∗*^*p* < 0.001.

**Figure 3:**
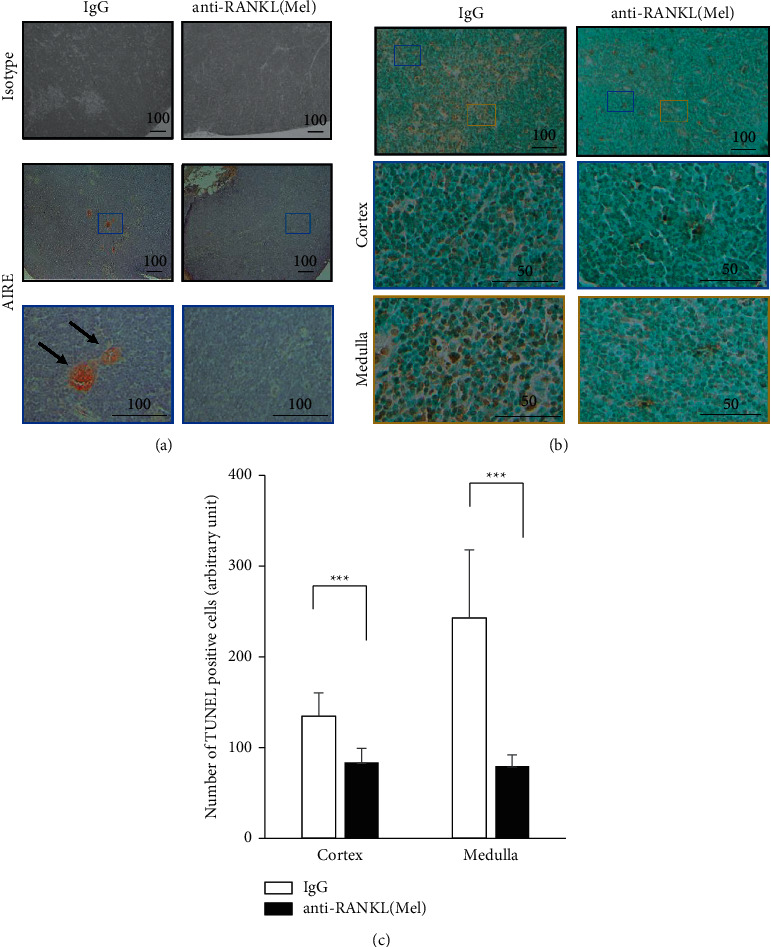
Immunochemical examination and TUNEL assay of the thymus. (a) The upper panels show an immunohistochemical image in the thymus with isotype IgG, which is used as a negative control of IgG and anti-RANKL/Mel. The middle panels show immunohistochemistry of a low magnification image in the thymus stained with anti-AIRE of IgG and anti-RANKL/Mel. The lower panels show immunohistochemistry of a high magnification image in the thymus stained with anti-AIRE of IgG and anti-RANKL/Mel. Black arrows point to the AIRE positive cells. Bar is 100 *μ*m. (b) The upper panels show TUNEL reaction of a low magnification image in the thymus of IgG and anti-RANKL/Mel. Bar is 100 *μ*m. The middle panels show TUNEL reaction of a high magnification image in the cortex of IgG and anti-RANKL/Mel. The lower panels show TUNEL reaction of a high magnification image in the medulla of IgG and anti-RANKL/Mel. Bar is 50 *μ*m. (c) Number of TUNEL positive cells in the cortex and medulla of IgG vs. anti-RANKL/Mel. Graphs show mean ± SEM. ^*∗∗∗*^*p* < 0.001.

To investigate how the combined administration of anti-RANKL and Mel affects immune function, the thymus (a key immune organ) was studied morphologically, histologically, and immunologically. The thymus width diameter of IgG was 8.2 ± 0.8 mm, whereas the thymus width diameter of anti-RANKL/Mel was 5.3 ± 0.5 mm (Figures 2(a)c)a–c2(a2(c)). The reduction of thymic width diameter was also contracted in Mel single administered mice and anti-RANKL single administered mice. Mel single administered mice had a thymic width of 5.8 ± 0.2 mm (*P* < 0.001), whereas anti-RANKL single administered mice had a thymic width of 6.7 ± 0.6 mm (*P* < 0.01), both of which were significantly atrophic compared with IgG (Appendix Figure 4). To determine where thymic atrophy occurred, the areas of cortex and medulla of the thymus were measured on sections. This resulted in a reduction in cortex from 58.7 ± 23.2 mm2 to 20.8 ± 12.4  mm2 to a size of approximately 30% (*P* < 0.001) (Figure 2(d)). On the other hand, in the medulla, it was reduced from 11.4 ± 3.7 mm2 to 2.4 ± 1.0 mm2 (*p* < 0.001) (Figure 2(d)). In anti-RANKL/Mel, the medulla was markedly reduced, and it was proven that the administration of anti-RANKL/MEL had more effects on the medulla. TUNEL staining of thymic tissue was performed to determine how anti-RANKL/Mel affects thymic function. Anti-RANKL/Mel had 79.6 ± 12.3 TUNEL positive cells in the medulla. This was significantly reduced compared to 243.0 ± 74.9 cells in IgG (*P* < 0.001) (Figures 3(b) and 3(c)). Additionally, there were 83.7 ± 15.6 TUNEL positive cells in the cortex in anti-RANKL/Mel. This was significantly reduced compared to 134.8 ± 25.5 cells in IgG (*P* < 0.001) (Figures 3(b) and 3(c)).

**Figure 4:**
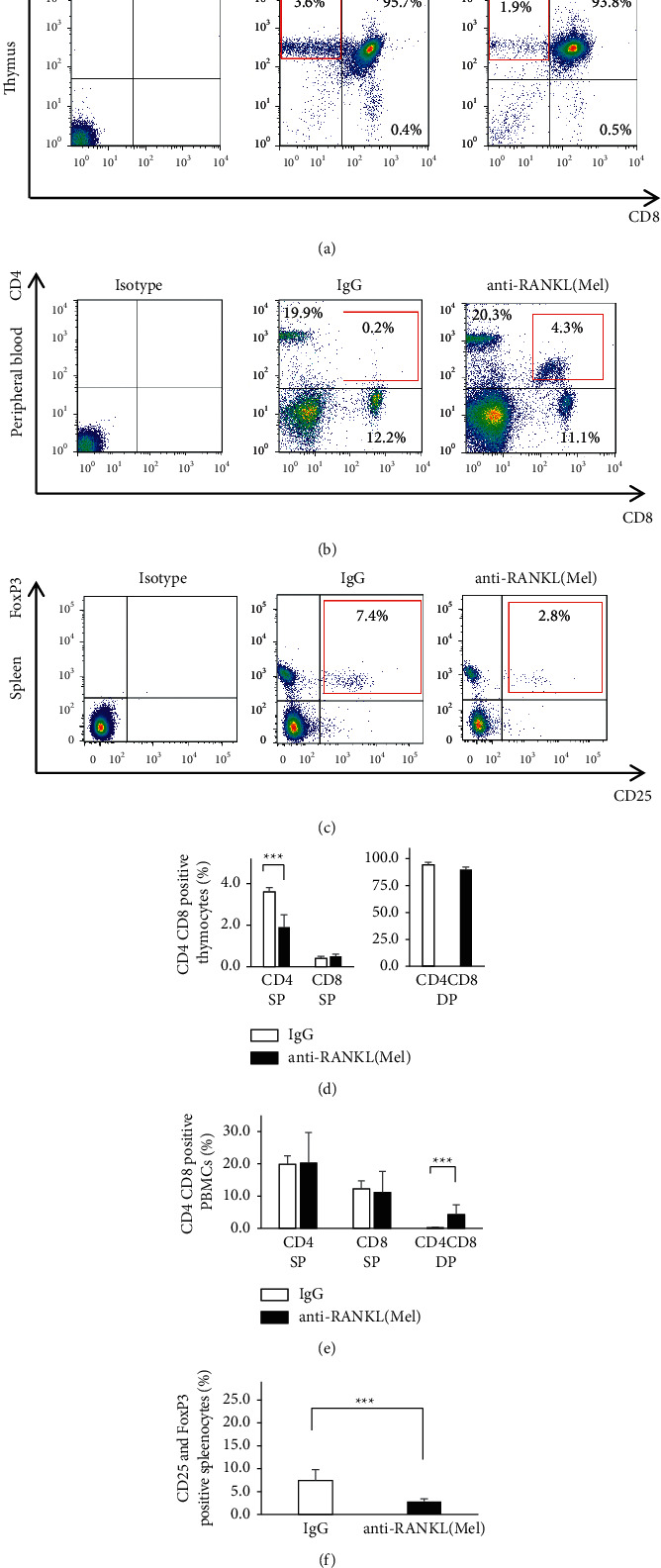
Flow cytometry analysis of thymocytes, PBMCs, and splenocytes. (a) Flow cytometric analysis of thymocytes from IgG and anti-RANKL/Mel. Thymocytes were analyzed for CD4 and CD8 expression. The numbers indicate the percent of total thymocytes that fall within the indicated quadrants. (b) Flow cytometric analysis of PBMCs from IgG and anti-RANKL/Mel. Thymocytes were analyzed for CD4 and CD8 expression. The numbers indicate the percent of total thymocytes that fall within the indicated quadrants. (c) Flow cytometric analysis of splenocytes from IgG and anti-RANKL/Me. Splenocytes were analysed for CD4 and CD8 expression. The numbers indicate the percent of total thymocytes that fall within the indicated quadrants. (d) Percent of CD4 SP, CD8 SP, and CD4CD8 DP cells in IgG and anti-RANKL/Mel. (e) Percent of CD4 SP, CD8 SP, and CD4CD8 DP cells in IgG and anti-RANKL/Mel mice. (f) Percent of CD25 and FoxP3 positive Treg cells in and anti-RANKL/Mel. Graphs show mean ± SEM. ^*∗∗∗*^*p* < 0.001.

In order to examine the treatment effects on immunity, T cells were isolated from the thymus, peripheral blood, and spleen four weeks after tooth extraction. Flow cytometry was performed using thymocytes and PBMCs, and the expression of CD4 and/or CD8 cells was quantified. The percentage of CD4+CD8 single positive T cells (CD4 SP) in the thymus was significantly decreased in anti-RANKL/Mel compared to IgG, 3.6 ± 0.2% in IgG and 1.9 ± 0.6% in anti-RANKL/Mel (*P* < 0.001) (Figures 4(a) and 4(d)). The percentage of CD4-CD8+SP T cells (CD8 SP) was not significantly different from 0.4 ± 0.1% in IgG and 0.5 ± 0.1% in anti-RANKL/Mel (Figures 4(a) and 4(d)). The percentage of CD4+ CD8+ double positive T cells (CD4CD8 DP) was not significantly different from 95.7 ± 0.3% for IgG and 93.8 ± 1.7% for anti-RANKL/Mel (Figures 4(a) and 4(d)). On the other hand, the percentage of CD4 SP in peripheral blood was not significantly different from 19.9 ± 2.6% in IgG and 20.3 ± 9.4% in anti-RANKL/Mel (Figures 4(b) and 4(e)). The percentage of CD8 SP was not significantly different from 12.2 ± 2.4% in IgG and 11.1 ± 6.5% in anti-RANKL/Mel (Figures 4(b) and 4(e)). However, the percentage of CD4CD8 DP was significantly increased in anti-RANKL/Mel compared to IgG, 0.2 ± 0.1% in IgG and 4.3 ± 3.0% in anti-RANKL/Mel (*P* < 0.001) (Figures 4(b) and 4(e)). We examined CD4+CD25+Foxp3+regulatory T cells (Treg) in the spleen. The percentage of Treg cells was 7.4 ± 2.4% in IgG compared to 2.8 ± 0.5% in anti-RANKL/Mel. This indicates that Treg were significantly reduced in anti-RANKL/Mel (*P* < 0.001) (Figures 4(c) and 4(f)).

## 4. Discussion

Animal models are key tools in addressing the disease pathogenesis of DRONJ. Therefore, several mouse models of DRONJ have been reported. These papers have been devised to induce DRONJ by administering large doses of anti-RANKL or combining other drugs in addition to anti-RANKL. Williams et al. administered 250 *μ*g of anti-RANKL eight times for a total of 2 mg during the four weeks between the beginning of the medication and sacrifice [5]. Given that the denosumab dosage for treatment of bone lesions or solid tumor bone metastases caused by multiple myeloma in humans is 120 mg, assuming a weight of 25 g for the mice used in this experiment, the dosage of anti-RANKL administered to the mice in this experiment corresponded to a dosage approximately 30 to 40 times the human dosage. Hayano et al. also administered cyclophosphamide (CY), an alkylating agent, in addition to anti-RANKL to develop ONJ-like lesions [6]. The total dose of anti-RANKL administered in this study was approximately 0.4 mg, which is approximately 6 to 8 times the human dose. Interestingly, DRONJ-like lesions did not develop in a single administration of anti-RANKL or a single administration of CY. These results could indicate that CY plays a crucial role in the development of DRONJ. In this study, instead of CY, we used Mel, which is the same alkylating agent but has a more immunosuppressive effect. Single administration of anti-RANKL and single administration of Mel did not cause DRONJ-like lesions in mice, but combined administration of anti-RANKL and Mel caused DRONJ-like lesions in mice. Additionally, in a preliminary study, we compared the administration levels of anti-RANKL (0.1 mg/kg, 0.5 mg/kg, and 1.0 mg/kg) under a constant dosage of Mel (7 mg/kg). We found that the incidence of DRONJ-like lesions increased in a concentration-dependent manner in anti-RANKL (data not shown). The administration protocol of this study is an important analytical model for clarifying the pathogenesis of DRONJ because DRONJ-like lesions are contracted by the administration of anti-RANKL in a dosage close to that used for human.

We considered that the lowering of immune function and the motion body of the immune cells in anti-RANKL/Mel was searched. Interestingly, the size of the thymus was confirmed to be atrophic in anti-RANKL/Mel (Figure 2). The thymus was also atrophic in anti-RANKL single administered mice and Mel single administered mice. However, when both were compared, atrophy of the thymus was more pronounced in Mel single administered mice (Appendix Figure 4). Interestingly, it was found that the thymus was atrophic in a concentration-dependent manner of anti-RANKL at constant concentrations of Mel (Appendix Figure 4). These findings suggest that the marked thymic atrophy occurring in anti-RANKL/Mel is a result of the myelosuppression induced by Mel. Additionally, anti-RANKL may act synergistically. The thymus is a primary lymphoid organ of the immune system that functions as a developmental tissue for T cells. In the cortex, the earliest T cells express neither CD4 nor CD8, and are classified as CD4-CD8-DN T cells (DN cells) [7]. CD4CD8 DN results in rearrangement of the TCR*β* gene in the thymic cortex and differentiates into CD4CD8 DP T cells (DP cells). DP cells are located in the cortex where the action of the cortical thymic epithelial cell (cTEC) removes T cells unresponsive to the major histocompatibility complex MHC (positive selection) [8]. DP cells that remain by positive selection migrate into the medulla but contain many autoreactive T cells that respond strongly to autologous cells. Therefore, autoantigen-reactive T cells are eliminated (negative selection) by the action of medulla thymic epithelial cells (mTECs) in the medulla. T cells that have undergone negative selection acquire central tolerance to self-antigens [9]. Disruption of central tolerance within the thymic medulla leads to autoimmune disease because autoreactive T cells are not cleared in the thymus. It is important that self-reactive T cells that bind strongly to self-antigens are eliminated in the thymus. T cell self-tolerance is established by mTEC through the expression of AIRE and plays a key role in imposing tolerance on negative selection [10–12]. Loss of AIRE or functional mutations in humans has been reported to lead to the development of autoimmune diseases, such as autoimmune polyendocrinopathy-candidiasis ectodermal dystrophy and polyglandular syndrome type 1 [13,14]. It has also been reported that loss of AIRE leads to systemic autoimmune manifestations similar to those in humans [15]. Interestingly, AIRE is activated in mTEC through the RANK signal, and both CD40 and RANK have been shown to play important roles in the generation of mTEC [16]. Several studies have investigated the cellular sources of RANKL and CD40 L in relation to immature T cell crosstalk and thymic medulla formation [17]. The absence of RANK expression leads to a marked reduction in the frequency of AIRE in both the fetal and adult thymus [18]. RANK deficiency and RANK/RANKL double deficiency also result in the onset of mediated autoimmunity [19]. Additionally, it has been reported that the thymus is enlarged in transgenic mice with AIRE [20]. Thus, administration of anti-RANKL is likely to result in decreased expression of AIRE and thymic atrophy.

Indeed, in anti-RANKL/Mel, the thymus was atrophic (Figure 2). It was proven that in the thymus of anti-RANKL/Mel, there was only slight expression of AIRE positive cells. This reduction in the numbers of AIRE positive cells may have been caused by the administration of anti-RANKL blocking RANK/RANKL signal between immature T cells and mTEC. The function of AIRE is to kill autoreactive T cells by apoptosis. Therefore, we examined how the number of cells undergoing apoptosis in the thymus was altered. Many TUNEL positive cells were identified in the IgG, whereas TUNEL positive cells were significantly reduced in anti-RANKL/Mel. This may indicate that the absence of AIRE does not eliminate autoreactive T cells. Next, the distribution of T cells in the peripheral blood was examined, and CD4CD8 DP was confirmed in the peripheral blood of anti-RANKL/Mel. Since CD4CD8 DP are not detected in IgG, CD4CD8 DP present in the peripheral blood seem to be autoantigen-reactive T cells that should be eliminated by negative selection of AIRE in the medulla. In a previous report, peripheral CD4CD8 DP has been reported to play a role in several autoimmune diseases and chronic inflammatory disorders. For example, CD4CD8 DP has been reported to be found at high levels in the peripheral blood of individuals with multiple sclerosis, which is an autoimmune disease [21]. CD4CD8 DP are increased in the periphery of people with severe rheumatoid arthritis [22]. The presence of CD4CD8 DP in these diseases suggests their involvement in their development. On the other hand, Treg cells suppress the immune response and are responsible for suppressing excessive immune responses that lead to autoimmune, inflammatory, and allergic diseases, thus playing a role in immune tolerance in peripheral tissues. The lack of AIRE in a mouse model is accompanied by defects in thymocyte development. Interaction with mTEC expressing AIRE in the thymus has been reported as critical for the maturation and subsequent stability of the phenotype and suppressive activity of Treg cells [23,24]. In this study, the number of Treg cells in the spleen of anti-RANKL/Mel was significantly decreased compared with that of IgG. We speculate that this reduction in Treg cell numbers is caused by a reduction in AIRE positive cells in the thymus. We speculate that the reduction of Treg cells in the spleen implies a disruption of peripheral tolerance. These findings suggest that the development of DRONJ may be caused by a breakdown of peripheral immune tolerance because of reduced expression of AIRE in the thymus.

## 5. Conclusion

We created a mouse disease model that was very similar to DRONJ in humans. Our results suggest that an imbalance of immune responses is a pathogenic mechanism of DRONJ. Although the pathophysiology of DRONJ may be complex and is influenced by many factors, disruption of T cell development in the thymus plays a direct etiological role in DRONJ. To the best of our knowledge, this is the first experimental study to illustrate that immune abnormalities of the T cell population are implicated in the pathogenic mechanisms of DRONJ. In humans, the immune dynamics of people who develop DRONJ should be investigated, but the risk of developing DRONJ may increase when teeth are extracted in the presence of abnormal immune function. This study will permit the development of therapeutic strategies and agents to treat human DRONJ.

## Data Availability

The data used in this study are available upon request.

## Supplementary Materials

Supplementary Materials Appendix Table: summary of clinical and histologic findings in mice of IgG administration, anti-RANKL/Mel administration, and single Mel administration. Appendix Figure 1: four weeks after tooth extraction, mice were treated with anti-RANKL under constant dosage of Mel. Appendix Figure 2: necrotic bone area of IgG administration, anti-RANKL/Mel administration, and single Mel administration. Appendix Figure 3: number of empty lacunae of necrotic bone area of IgG administration, anti-RANKL/Mel administration, and single Mel administration. Appendix Figure 4: wide length of thymus with IgG administration, anti-RANKL/Mel administration, and single Mel administration.
