# Supplementary material for: Anti-RANKL Inhibits Thymic Function and Causes DRONJ in Mice
Source: Int J Dent. 2022 Apr 15;2022:9299602. doi: 10.1155/2022/9299602 (PMC9033356; doi:10.1155/2022/9299602)
Supplement: Supplementary Materials — Appendix Table: summary of clinical and histologic findings in mice of IgG administration, anti-RANKL/Mel administration, and single Mel administration. Appendix Figure 1: four weeks after tooth extraction, mice were treated with anti-RANKL under constant dosage of Mel. Appendix Figure 2: necrotic bone area of IgG administration, anti-RANKL/Mel administration, and single Mel administration. Appendix Figure 3: number of empty lacunae of necrotic bone area of IgG administration, anti-RANKL/Mel administration, and single Mel administration. Appendix Figure 4: wide length of thymus with IgG administration, anti-RANKL/Mel administration, and single Mel administration. [file 9299602.f1.docx]

Appendix Table and Figures

Appendix Table


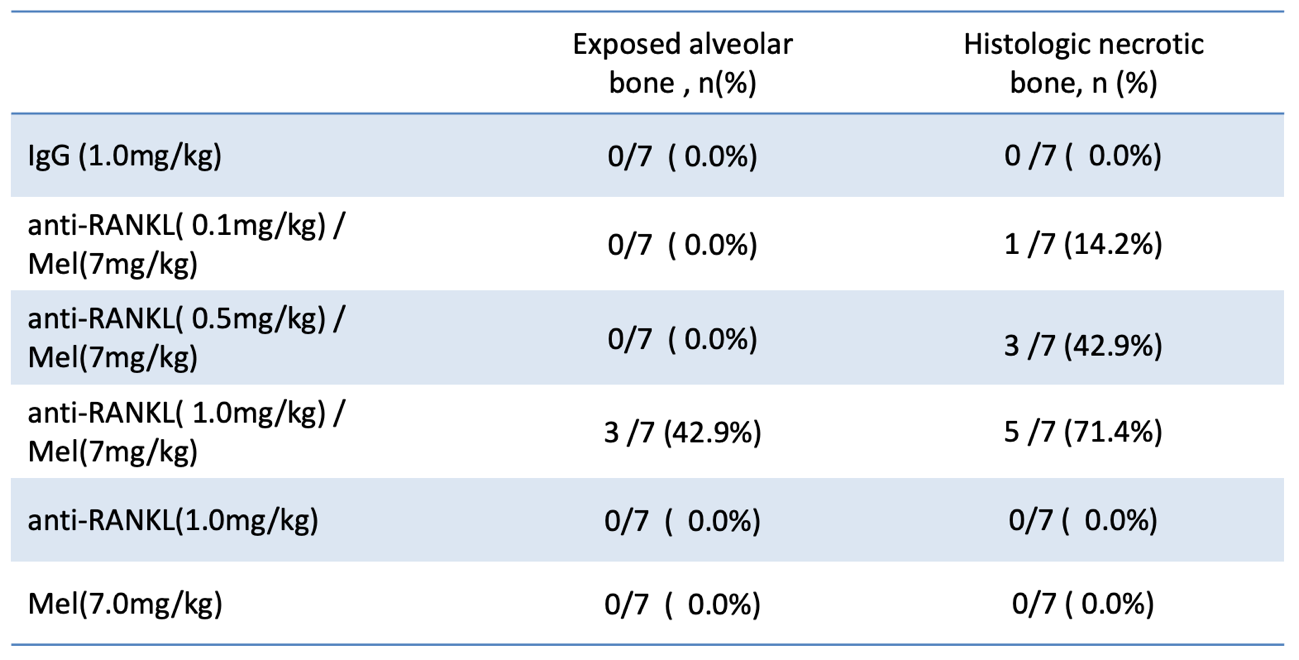


Summary of clinical and histologic findings in mice of IgG administration, anti-RANKL (0 mg/kg, 0.1 mg/kg, 0.5 mg/kg, 1.0 mg/kg) /Mel (7 mg/kg) administration and single Mel (7 mg/kg) administration.

Appendix Figure 1


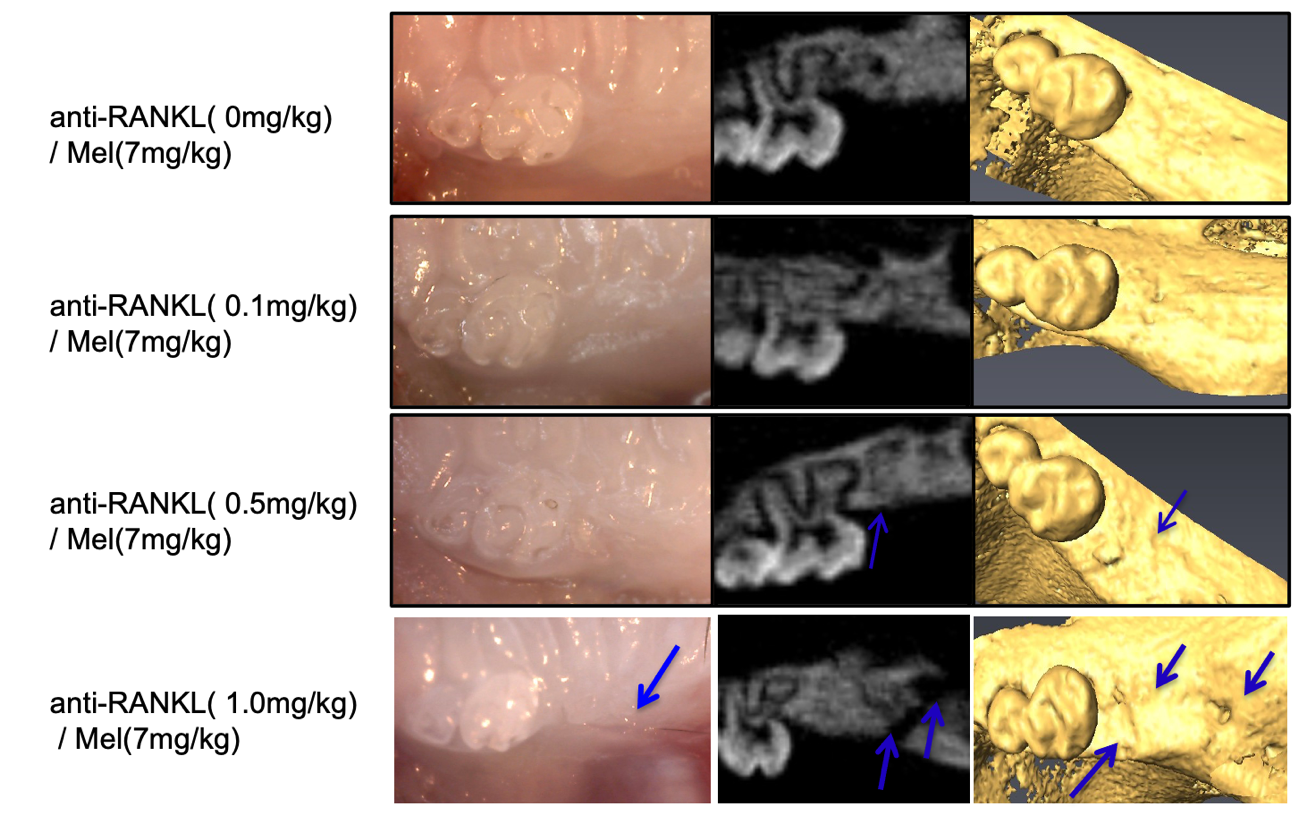


Four weeks after tooth extraction, mice were treated with anti-RANKL (0 mg/kg, 0.1 mg/kg, 0.5 mg/kg, 1.0 mg/kg) under constant dosage of Mel (7 mg/kg). Representative gross appearance of gingival mucosa, site μCT scans image and 3D image at the extraction of the extraction site. Blue arrows indicate extraction sockets of the root.

Appendix Figure 2


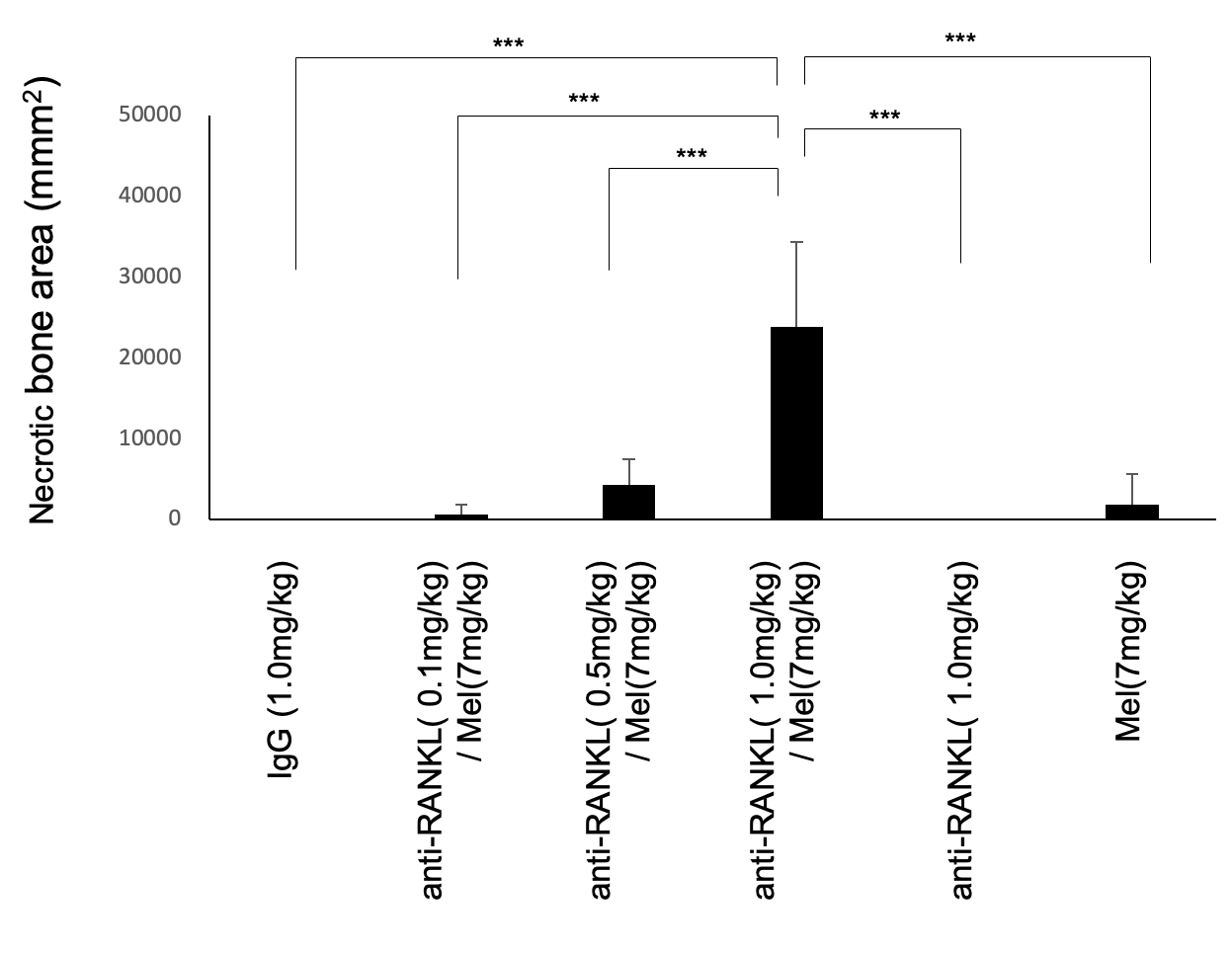


Necrotic bone area of IgG administration, anti-RANKL (0 mg/kg, 0.1 mg/kg, 0.5 mg/kg, 1.0 mg/kg) /Mel (7 mg/kg) administration and single Mel (7 mg/kg) administration.

Graphs show mean ± SEM. ***p < 0.001

Appendix Figure 3


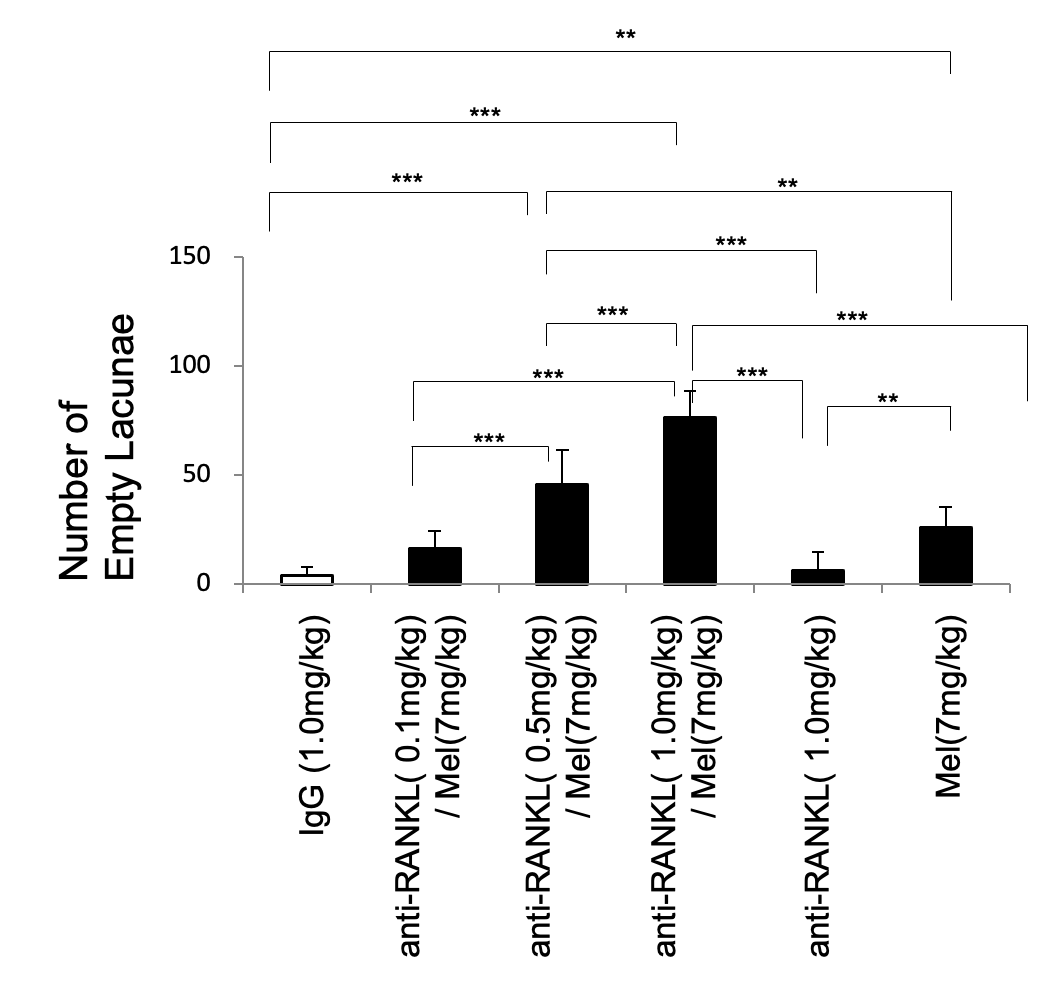


Number of empty lacunae of necrotic bone area of IgG administration, anti-RANKL (0 mg/kg, 0.1 mg/kg, 0.5 mg/kg, 1.0 mg/kg) /Mel (7 mg/kg) administration and single Mel (7 mg/kg) administration. Graphs show mean ± SEM. **p < 0.01, ***p < 0.001

Appendix Figure 4


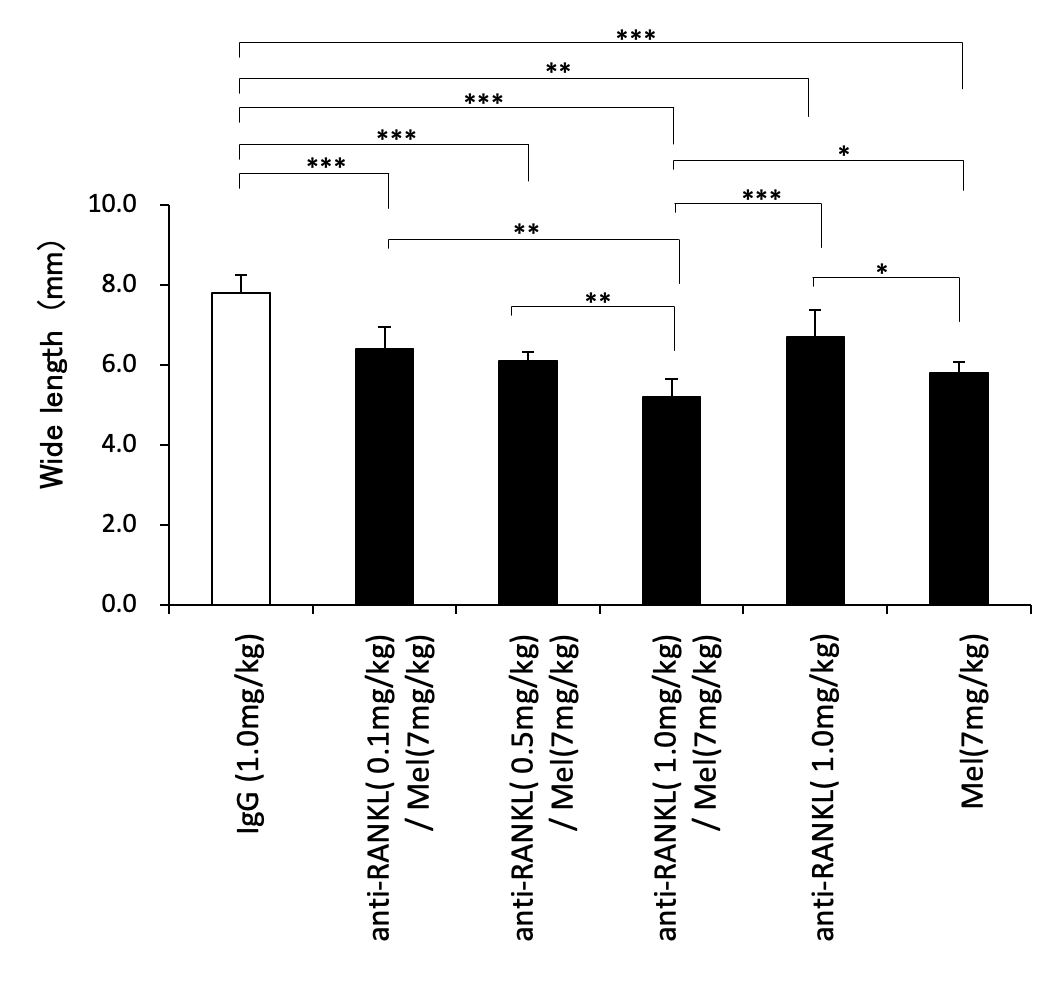


Wide length of thymus with IgG administration, anti-RANKL (0 mg/kg, 0.1 mg/kg, 0.5 mg/kg, 1.0 mg/kg) /Mel (7 mg/kg) administration and single Mel (7 mg/kg) administration. Graphs show mean ± SEM. *p < 0.05, **p < 0.01, ***p < 0.001
